# The microbiology of diabetic foot infections: a meta-analysis

**DOI:** 10.1186/s12879-021-06516-7

**Published:** 2021-08-09

**Authors:** Katherine E. Macdonald, Sophie Boeckh, Helen J. Stacey, Joshua D. Jones

**Affiliations:** 1 Infection Medicine, Edinburgh Medical School: Biomedical Sciences, University of Edinburgh, Chancellor’s Building, 49 Little France Crescent, Edinburgh, EH16 4SB UK; 2 Faculty of Medicine and Health Sciences, McGill University, 3605 Rue de la Montagne, Montreal, QC H3G 2M1 Canada; 3 Edinburgh Medical School, University of Edinburgh, Chancellor’s Building, 49 Little France Crescent, Edinburgh, EH16 4SB UK

**Keywords:** Diabetes, Diabetic foot infection, Diabetic foot ulcer, Meta-analysis, Microbiology, Prevalence

## Abstract

**Background:**

Diabetic foot ulcers are a common complication of poorly controlled diabetes and often become infected, termed diabetic foot infection. There have been numerous studies of the microbiology of diabetic foot infection but no meta-analysis has provided a global overview of these data. This meta-analysis aimed to investigate the prevalence of bacteria isolated from diabetic foot infections using studies of any design which reported diabetic foot infection culture results.

**Methods:**

The Medline, EMBASE, Web of Science and BIOSIS electronic databases were searched for studies published up to 2019 which contained microbiological culture results from at least 10 diabetic foot infection patients. Two authors independently assessed study eligibility and extracted the data. The main outcome was the prevalence of each bacterial genera or species.

**Results:**

A total of 112 studies were included, representing 16,159 patients from which 22,198 microbial isolates were obtained. The organism most commonly identified was *Staphylococcus aureus*, of which 18.0% (95% CI 13.8–22.6%; I^2^ = 93.8% [93.0–94.5%]) was MRSA. Other highly prevalent organisms were *Pseudomonas* spp., *E. coli* and *Enterococcus* spp. A correlation was identified between Gross National Income and the prevalence of Gram positive or negative organisms in diabetic foot infections.

**Conclusion:**

The microbiology of diabetic foot infections is diverse, but *S. aureus* predominates. The correlation between the prevalence of Gram positive and negative organisms and Gross National Income could reflect differences in healthcare provision and sanitation. This meta-analysis has synthesised multiple datasets to provide a global overview of the microbiology of diabetic foot infections that will help direct the development of novel therapeutics.

**Supplementary Information:**

The online version contains supplementary material available at 10.1186/s12879-021-06516-7.

## Background

Diabetes mellitus, herein diabetes, is a major global health issue, affecting an estimated 382 million people worldwide. The global prevalence of diabetes is rising, and by 2035 approximately 592 million people will be affected [1]. Poorly controlled diabetes can predispose patients to diabetic foot ulcers (DFUs). It has been estimated that 15% of diabetics will develop a DFU in their lifetime [2]. The costs associated with DFU care are substantial. In 2014–2015 the annual cost of care for diabetic foot in England was estimated to be around £1 billion [3].

The aetiology of DFUs typically reflects trauma superimposed upon peripheral neuropathy and ischaemia. Such diabetic foot ulcers commonly become sources of intransigent infection, whereupon they may be termed diabetic foot infections (DFIs). Although initially superficial, DFIs can become complicated by osteomyelitis [4]. Management of DFIs is limited to wound care, antibiotics and amputation [5]. These infections can be difficult to treat and, despite the administration of multiple rounds of antibiotics, prospects of clinical resolution of infection can still be poor and repeated courses of antibiotics risks selecting for antimicrobial resistance. A recent study in the United Kingdom found that one year after diagnosis, 55% of DFI patients were still infected and almost 15% had undergone amputation [6]. The treatment of DFIs therefore represents a significant clinical challenge.

An understanding of the microbiology of DFIs is key to tackling this clinical challenge. The microbiology of DFIs infections has been thoroughly investigated by prospective and retrospective studies. A diverse range of pathogens may be isolated from DFIs, reflecting the chronic, open, nature and anatomical location of these infections [7]. DFIs can be either mono- or polymicrobial, with polymicrobial being common among chronic infections that have undergone previous antibiotic treatment [8]. Gram positive cocci, particularly *Staphylococci*, are frequently isolated [9]. However, Gram negative organisms including *Enterococcus faecalis*, *Enterobacter cloacae* and *Proteus mirabilis* have also been observed at notable frequencies [10]. Deeper DFIs have been shown by some studies to be associated with the presence of anaerobic organisms [11]. Organisms with antimicrobial resistance are commonly found in DFIs, potentially reflecting the extent of patient interaction with healthcare environments or because of exposure to repeated courses of antibiotics [12]. Moreover, bacteria frequently form biofilms that resist immune clearance and promote antimicrobial resistance [13]; in one study 78.2% of chronic wounds showed evidence of biofilm production [14].

A greater understanding of the microbiology of DFIs is important to help inform antimicrobial therapy and direct the development of novel therapeutics. While there have been many small or moderate scale investigations, to date no meta-analysis has sought to examine the global prevalence of the different microbes identified. Therefore, the aim of this meta-analysis was to use diagnostic studies of any design which reported data for 10 or more DFI culture results to investigate the prevalence of bacteria isolated from DFIs.

## Methods

### Search strategy

Four electronic databases were searched for articles published from 1980 up to and including 2019: EMBASE (1980–2019), Ovid MEDLINE® Epub Ahead of Print, In-Process & Other Non-Indexed Citations, Ovid MEDLINE® Daily, Ovid MEDLINE and Versions® (1946–2019), Web of Science and BIOSIS Citation Index (1926–2019). The Web of Science Core Collection Citation Indexes searched were: Science Citation Index Expanded (1900–2019), Book Citation Index-Science (2005–2019) and the Emerging Sources Citation Index (2015–2019). The search was performed using the following terms: (diabe* adj2 [foot or toe]) AND (osteo* OR ulcer* OR lesion* OR infect*) AND (culture* OR microbio* OR bacteria* OR isolate* OR pathogen$ OR sequenc* OR swab*). In Ovid these terms were followed by the suffix ‘.mp.’ and they were searched as topics in Web of Science. A study protocol was not published prior to this study.

### Study selection criteria

All studies underwent title and abstract screening, eligible studies met the following criteria: (1) the patients had been diagnosed with diabetes mellitus and a diabetic foot infection; (2) primary microbiological culture results were collected from foot swabs or biopsies; (3) it was clearly stated or could be reasonably inferred that one clinical sample was obtained per patient, data presented as percentages were excluded if they could not confidently be converted into raw data; (4) microbiological prevalence data had to be presented in full; (5) the study was published in the English language; (6) to minimize selection bias from individual case reports or short case series, studies had to contain data for 10 or more patients. There were no limitations on study type or location. For studies which presented the results of both superficial and deep sampling methods for all patients only the data from the deep sampling method was included. Where there was no mention of culture negative clinical samples there were assumed to be none, unless otherwise reasonably implied. Studies were excluded if they contained any discrepancy between summary values and raw data. Where studies provided antibiotic sensitivity data but did not explicitly report the frequency of MRSA, we used evidence of *S. aureus* resistance to methicillin or oxacillin to deduce the frequency of MRSA. The term ‘diphtheroid’ is synonymous with *Corynebacterium* and data from both was combined under the latter heading.

Eligible studies were accessed in full to ensure they fulfilled the inclusion criteria and provided sufficient data for the meta-analysis. Studies which could not be accessed in full, including presentation abstracts and articles behind paywalls, were excluded. A few studies required additional interpretation. One study referred to ‘*Staphylococcus*: COP’, interpreted as coagulase-positive *S. aureus* (15). Another study referred to ‘*Streptococcus aureus*’, despite showing *Streptococcus* as a separate column; this was also assumed to represent *S. aureus* [16]. A further study referred to ‘central nervous system’, interpreted as CNS or coagulase-negative Staphylococci [17].

Title and abstract and full-text screening were performed independently by the authors (SB/KM, JDJ), with discrepancies resolved by agreement or, where necessary, a third author (HJS). Deduplication was performed using Endnote (version X8.0.1). This review was conducted in accordance with the PRISMA (Preferred Reporting Items for Systematic Reviews and Meta-Analyses) guidelines [18], and a PRISMA checklist completed (see Additional file 1).

### Data extraction and critical appraisal

The following information was extracted from each study into a spreadsheet: publication year; author(s); short citation; title; study design; study date(s); country; national income classification; whether anaerobes were tested for; whether patients on antibiotics were excluded; the number of patients cultured, samples, positive samples, samples without growth, samples with polymicrobial culture, samples with monomicrobial culture; the total number of microorganisms isolated; the frequency of bacterial genera or species isolated. Prevalence data were collected per bacterial genera, with the exceptions of *S. aureus* and *E. coli* which were consistently reported species. National income classification was based on Gross National Income classification from The World Bank [19]. Data extraction was performed independently by the authors (SB/KM, JDJ), with discrepancies resolved by agreement or, where necessary, a third author (HJS). All eligible studies were critically assessed using a modified Joanna Briggs Institute checklist for prevalence studies [20]. Critical appraisal was performed independently by two authors (SB, JDJ), with discrepancies resolved by a third author (HS). The influence of potential publication bias is addressed in the discussion.

### Statistical analysis

Random-effects meta-analyses were used throughout to calculate the pooled prevalence of the bacterial species identified with 95% confidence intervals (95% CIs). Study heterogeneity was assessed by the I^2^ statistic, reported with 95% CIs, and interpreted as low (≤ 25%), moderate (25–75%) or high (≥ 75%) [21]. All meta-analyses were carried out using MedCalc statistical software, version 19.8 (MedCalc Software, Ostend, Belgium). The association between the study dates and bacterial prevalence was assessed by linear regression, using Excel (version 2104; Microsoft Corporation, Seattle, United States). The significance of proportions was compared using the MedCalc N-1 Chi-squared calculator [22].

## Results

### Study selection and characteristics

**Fig. 1:**
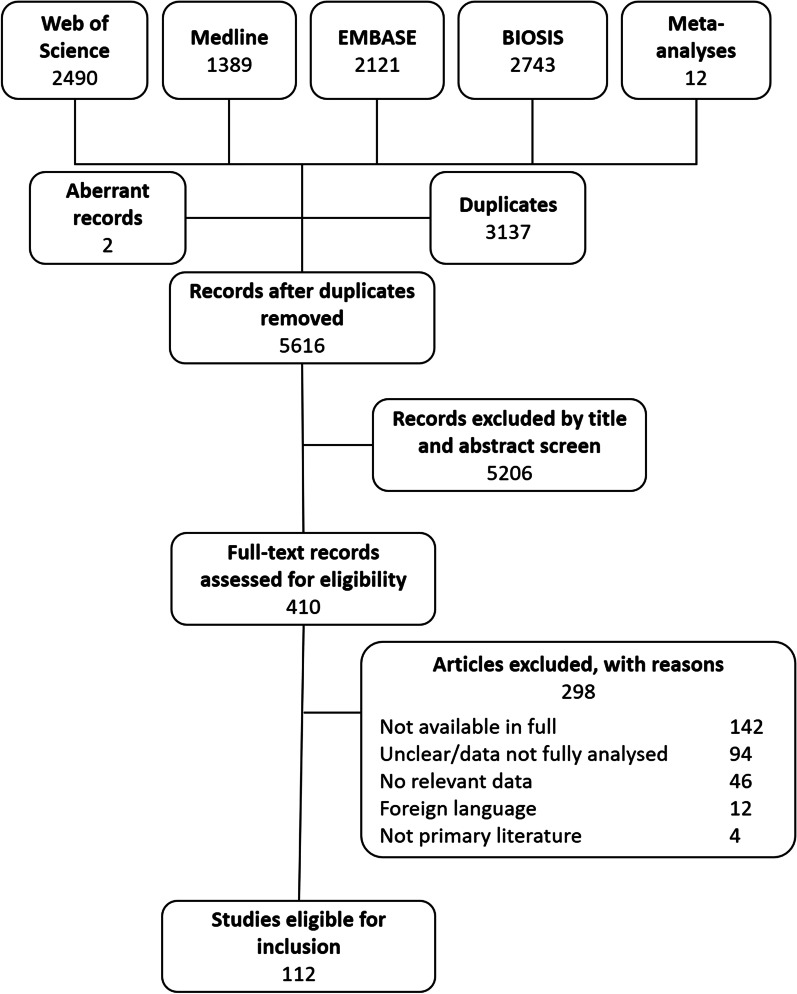
Flow diagram of study selection

Systematic searching of four databases yielded 8743 articles. Deduplication removed 3137 and two aberrant entries were removed, one was a corrupt entry and one published outside the search date range. The titles and abstracts of the remaining 5604 were screened against the selection criteria. A further 12 relevant articles were identified from related meta-analyses and also screened [23,24,25]. Title and abstract screening identified 410 eligible articles, 297 of which were subsequently excluded after full-text screening. Articles were excluded because they were not available in full (n = 142), unclear or did not present a full analysis of the data (n = 94), did not contain relevant data (n = 46), were not available in English (n = 12) or were not primary literature (n = 4). The study selection process is shown in Fig. 1. A total of 112 studies were eligible for inclusion (see Additional file 2). These comprised 76 prospective studies, 33 retrospective studies and three clinical trials. While critical appraisal of eligible studies highlighted shortcomings in reporting it did not reveal further grounds to exclude any studies (see Additional file 3).

Most of the 112 studies (n = 84) of the studies were published between 2011 and 2019, 25 took place between 2000 and 2010 and three were published before the year 2000, the earliest in 1984. The most frequent study locations were India (n = 32), Turkey (n = 8), China (n = 7) and France (n = 7). One study did not report the study location. Where reported, there were 42 study populations from high-income countries (HICs), 27 from upper middle-income countries (UMICs), 42 from lower-middle income countries (LMICs) and none from low-income countries. Patients that had recently been on antibiotics were excluded in 23 studies. Almost half (n = 55) of the studies tested for both anaerobic and aerobic bacterial species, while the remaining 57 studies tested only for aerobic bacteria.

The 112 studies contained microbiological prevalence data for 16,159 patients, of which 14,445 (89.4%) were positive for microbial growth. A total of 22,198 microbial isolates were obtained from these positive cultures; 1.54 isolates per culture. These were overwhelmingly bacterial, although 258 fungal isolates and one archaea were identified. The genus of most bacterial isolates was clearly reported, except for 231 unspecified Gram negative isolates, 127 unspecified Gram positive isolates, 416 members of the *Enterobacteriaceae* family and 141 unspecified isolates. Complete data about the frequency of negative culture, polymicrobial and monomicrobial results was available from 75 studies, representing 9737 patients. Of these, 8568 (88.0%) were culture positive, with 41.1% yielding monomicrobial growth and 58.9% yielding polymicrobial growth.

### A diverse range of microbial species is found in diabetic foot infections

The 112 datasets were used to determine the frequency of the organisms identified in DFIs. First, the studies were divided into two groups, those that tested for only aerobic isolates (n = 57) and those that tested for both aerobic and anaerobic isolates (n = 55).

**Fig. 2:**
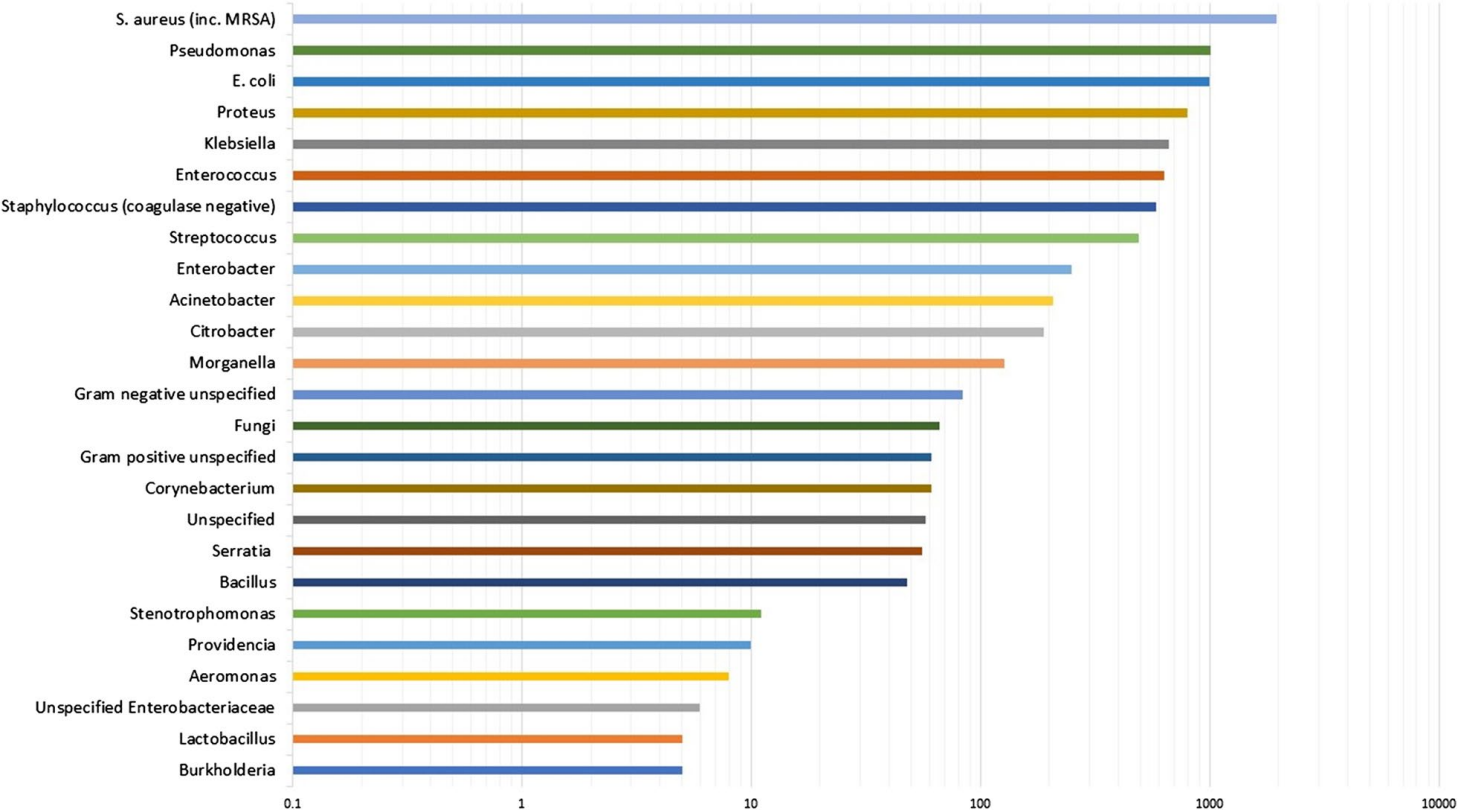
The frequency of microorganisms identified by aerobic culture of diabetic foot infection specimens

**Fig. 3:**
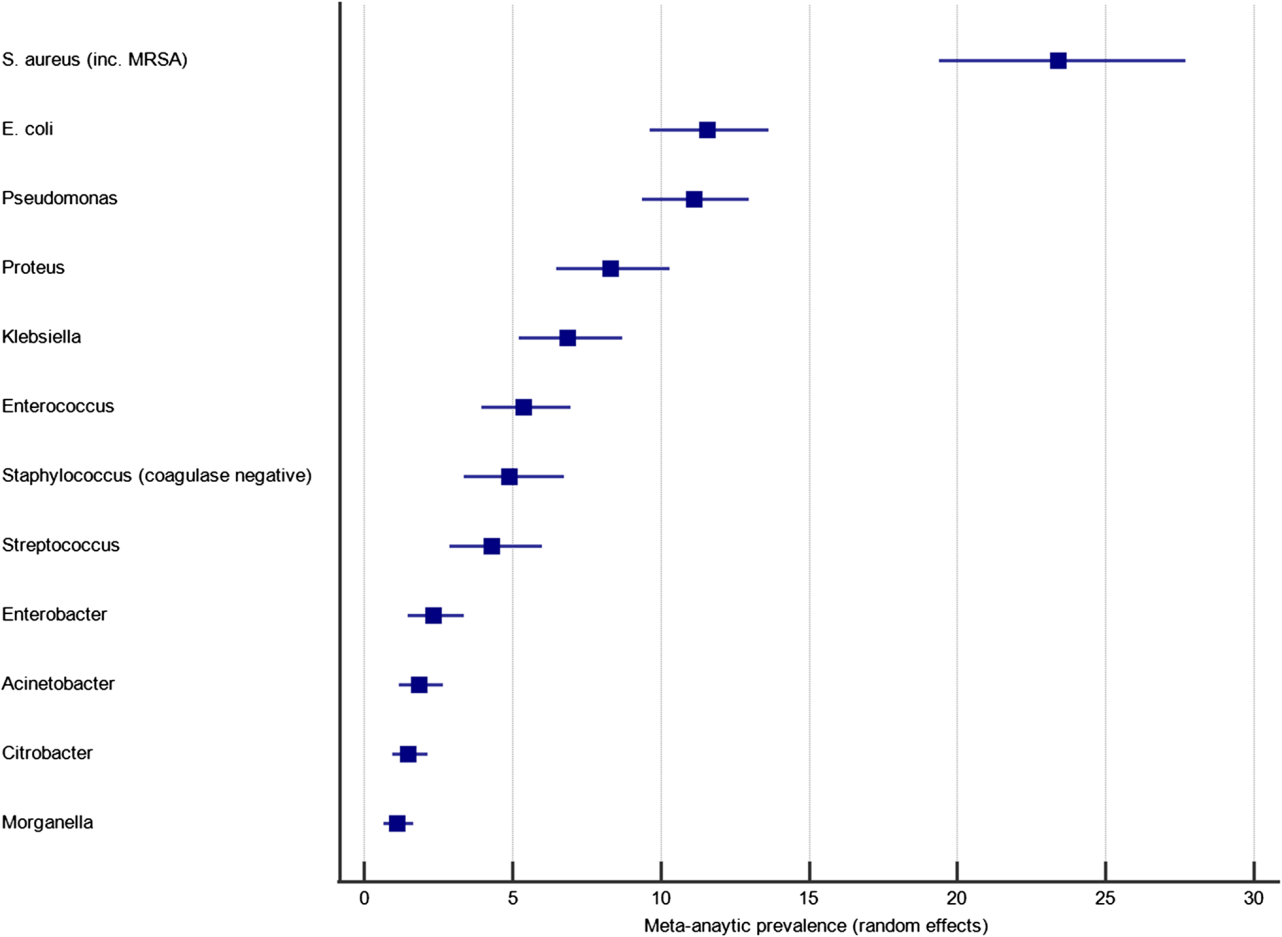
A forest plot of the meta-analytic prevalence of the microorganisms identified by aerobic culture of diabetic foot infection specimens. For clarity only genera with a prevalence > 1.0% are plotted, with prevalence data for genera < 1.0% available in Additional file 2

The 57 studies that tested for only aerobic growth represented 6736 clinical samples, of which 5945 (88.3%) were culture positive, yielding 8418 microbial isolates. The frequency of bacterial genera detected on five or more occasions is shown in Fig. 2. The three most frequently identified organisms were *Staphylococcus aureus, Pseudomonas* spp. and *E. coli*. To obtain a weighted average, meta-analyses were performed to investigate the pooled prevalence of each bacterial genus. These prevalence data are shown in the forest plot in Fig. 3. These meta-analyses show that the most frequently isolated aerobic organisms were *S. aureus* (23.4%; 95% CI 19.4–27.7%; I^2^ = 95.1% [94.3–95.8%]), *Pseudomonas* spp. (11.1%; 95% CI 9.4–13.0%; I^2^ = 85.0% [77.2–85.7%]), *E. coli* (11.5%; 95% CI 9.6–13.6%; I^2^ = 87.5% [84.6–89.9%]), *Proteus* spp. (8.3%; 95% CI 6.5–10.3%; I^2^ = 89.7% [87.4–91.6%]), *Klebsiella* spp. (6.9%; 95% CI 5.3–8.7%; I^2^ = 89.6% [87.3–91.4%]) and *Enterococcus* spp. (5.4%; 95% CI 4.0–7.0%; I^2^ = 88.8% [84.7–90.9%]).

**Fig. 4:**
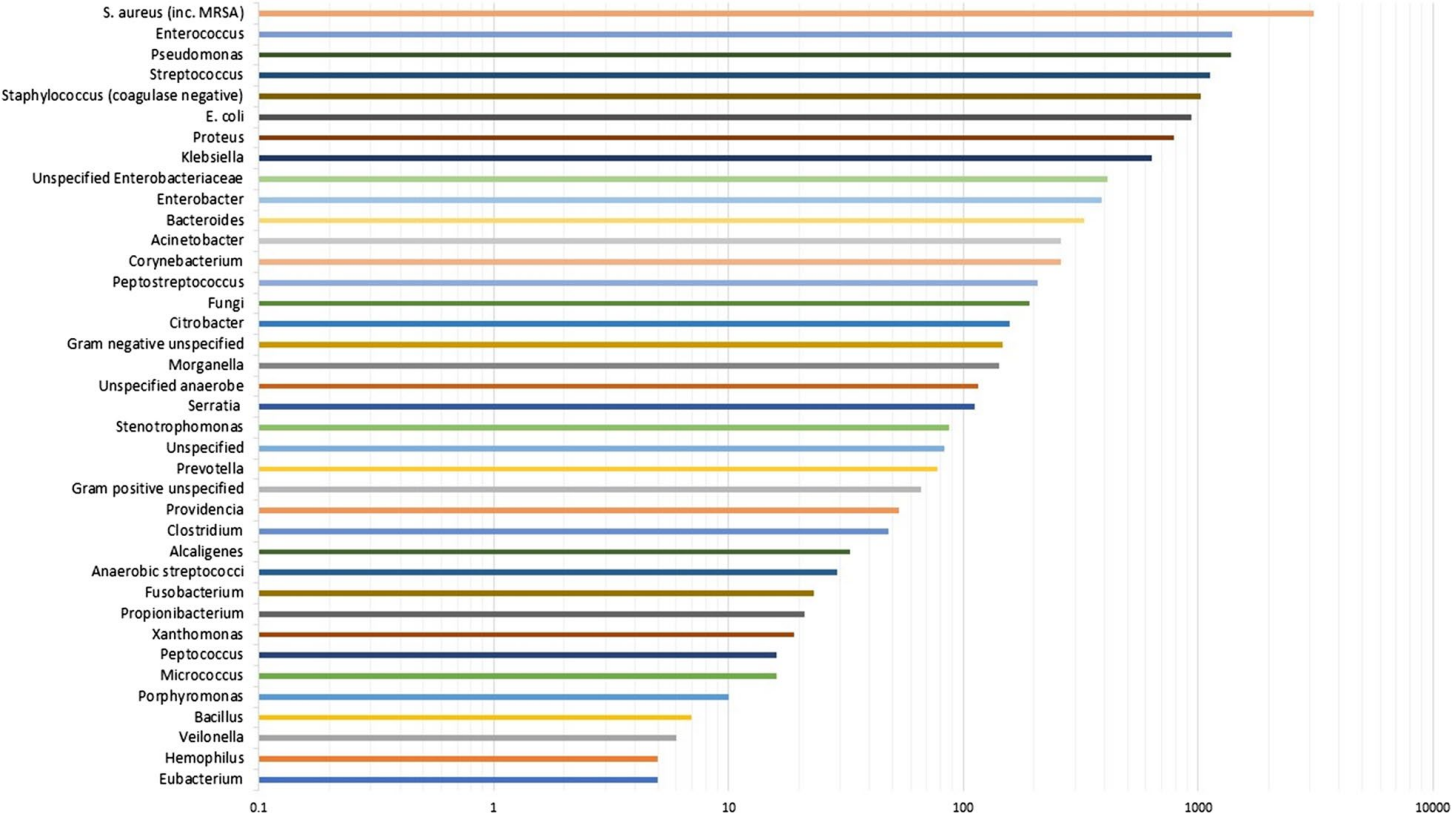
The frequency of microorganisms identified by aerobic or anaerobic culture of diabetic foot infection specimens

**Fig. 5:**
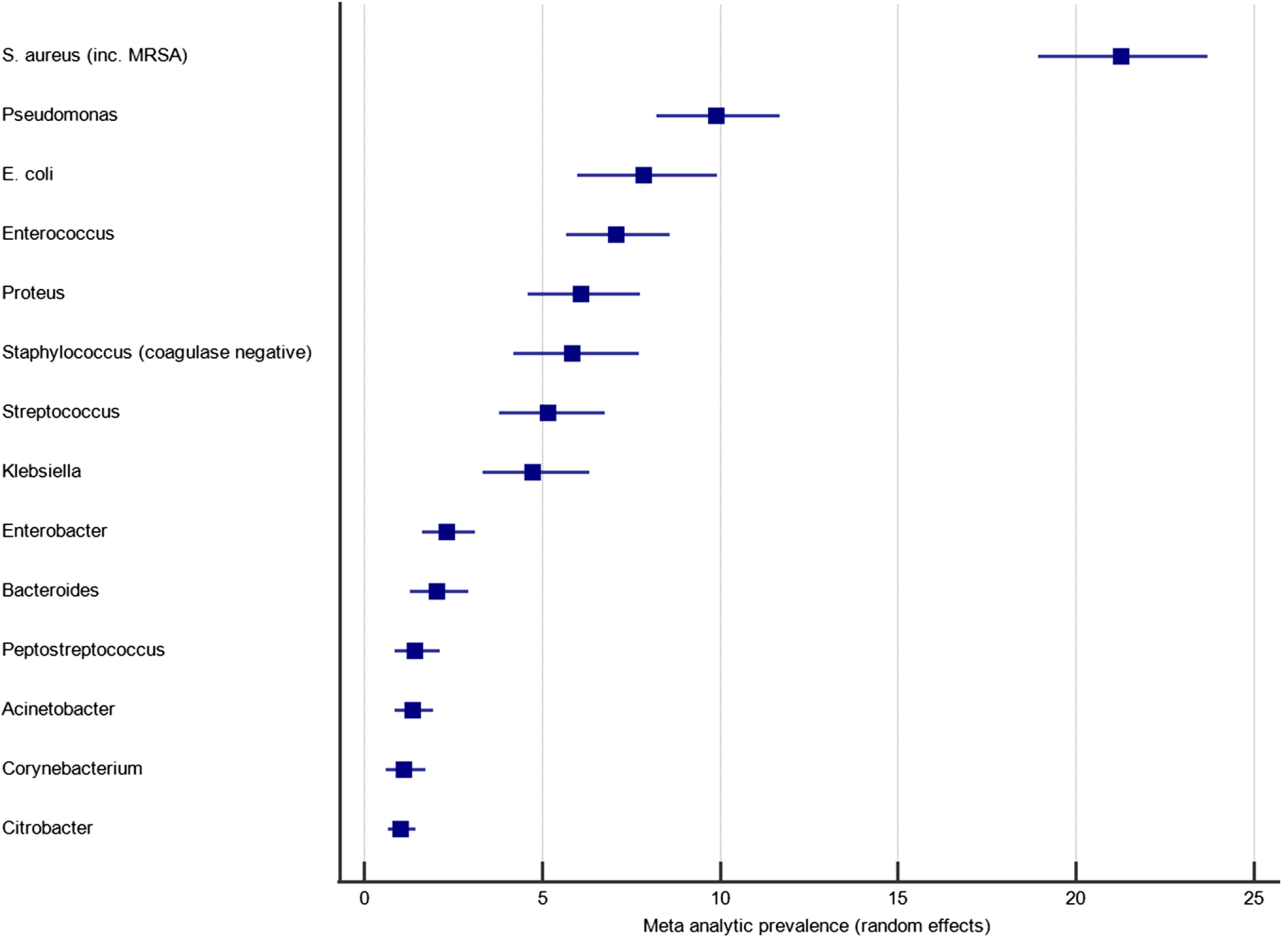
A forest plot of the meta-analytic prevalence of the microorganisms identified by aerobic or anaerobic culture of diabetic foot infection specimens. For clarity only genera with a prevalence > 1.0% are plotted, with prevalence data for genera < 1.0% available in Additional file 2

The 55 studies that tested for aerobic and anaerobic growth represented 9423 clinical samples, of which 8500 (90.2%) were culture positive, yielding 13,763 microbial isolates. The frequency of bacterial genera detected on five or more occasions is shown in Fig. 4. The three most frequently identified organisms were *Staphylococcus aureus, Enterococcus* spp. and *Pseudomonas* spp. As before, meta-analyses were performed to investigate the pooled prevalence of each bacterial genus. These prevalence data are shown in the forest plot in Fig. 5. These meta-analyses show that the most frequently isolated aerobic organisms are *S. aureus* (21.3%; 95% CI 18.9–23.7%; I^2^ = 91.0% [89.0–92.3%]), *Pseudomonas* spp. (9.9%; 95% CI 8.2–11.7%; I^2^ = 90.8% [88.9–92.5%]), *E. coli* (7.9%; 95% CI 6.0–9.9%; I^2^ = 94.3% [93.3–95.2%]), *Enterococcus* spp. (7.1%; 95% CI 5.7–8.6%; I^2^ = 90.3% [88.1–92.1%]), *Proteus* spp. (6.1%; 95% CI 4.6–7.8%; I^2^ = 93.1% [91.7–94.2%]), coagulase-negative *Staphylococci* (5.8%; 95% CI 4.2–7.7%; I^2^ = 94.7% [93.8–95.5%]) and *Streptococcus* spp. (5.2%; 95% CI 3.8–6.8%; I^2^ = 93.2% [91.9–94.3%]). Anaerobic organisms were not notably prevalent, with only *Bacteroides* spp. (2.0%; 95% CI 1.3–2.9%; I^2^ = 90.5% [88.4–92.2%]) and *Peptostreptococcus* spp. (1.4%; 95% CI 0.8–2.1%; I^2^ = 88.8% [86.2–90.9%]) occurring at a prevalence of greater than 1%.

### The prevalence of MRSA among DFIs

Diabetic patients are thought to have a higher chance of being colonized or infected by MRSA than non-diabetic patients [26, 27]. *S. aureus* isolates were detected in 109 of the 112 studies. A meta-analysis was used to investigate the proportion of *S. aureus* isolates that were reported as MRSA. The 109 studies included in this analysis represented 15,670 clinical samples, among which 5073 *S. aureus* isolates were obtained. The proportion of MRSA among these isolates was 18.0% (95% CI 13.8–22.6%; I^2^ = 93.8% [93.0–94.5%]). There was no correlation between year of publication and the prevalence of MRSA among *S. aureus* isolates (R^2^ = 0.0024; data not shown).

### Microbial prevalence correlates with gross national income

The 112 datasets included in this study were drawn from a wide range of countries, representing varying levels of healthcare provision and sanitation. It has been suggested that DFIs from less developed countries more often contain Gram negative organisms, with Gram positive organisms predominating among DFIs in more developed nations [28]. We therefore investigated whether the prevalence of aerobic Gram negative and Gram positive bacteria found in DFIs varied by Gross National Income (GNI). The 57 studies which used only aerobic culture were divided into two groups. The first group contained 13 studies from high-income countries (HICs), representing 1900 clinical samples from which 2317 isolates were obtained. The second group contained 43 studies from upper-middle and lower-middle income countries (U/LMICs), representing 4786 clinical samples from which 6051 isolates were obtained. There was one further study, the location of which was not reported.

The proportion of Gram positive and negative isolates was compared between studies from HICs and U/LMICs, excluding fungal, archaeal and unspecified isolates. The 13 studies from HICs reported 2273 isolates that were classified by Gram staining, of which 62.4% were Gram positive and 37.6% Gram negative. The 43 studies from U/LMICs reported 5970 isolates that were classified by Gram staining, of which 40.4% were Gram positive and 59.6% Gram negative. The 22.0% (95% CI 19.6–24.3%) difference between the proportion of Gram positive and negative isolates between U/LMICs and HICs was significant (P < 0.0001).

**Fig. 6:**
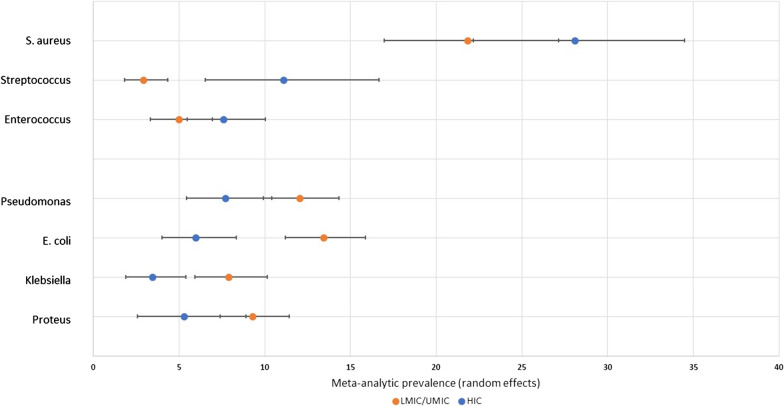
The meta-analytic prevalence of the bacteria most frequently identified by aerobic culture in high-income (HIC) or upper-middle and lower-middle income countries (U/LMIC)

The data from the above meta-analyses of the prevalence of bacterial genera were used to select the most commonly occurring Gram positive and Gram negative genera. The meta-analytic prevalence of these genera was calculated using the data from studies undertaken in HICs and U/LMICs. These data are shown in Fig. 6. The prevalence of Streptococcal species was significantly greater among HICs. The prevalence of *Enterococcus* spp. and *S. aureus* was also higher among HICs, but not significantly so. Among the Gram negative genera, *Klebsiella* spp. and *E. coli* were significantly more common among DFIs in U/LMICs than HICs. *Proteus* spp. and *Pseudomonas* spp. were also more prevalent among U/LMICs, but not significantly so. Finally, we compared the prevalence of MRSA reported in studies from HICs and U/LMICs. The 13 studies from HICs reported *S. aureus* isolates, with a total of 624, among which the prevalence of MRSA was 22.5% (95% CI 12.1–35.1%; I^2^ = 91.1% [86.6–94.1%]). Forty of the 43 studies from U/LMICs reported *S. aureus* isolates, with a total of 1314, among which the prevalence of MRSA was 19.2% (95% CI 11.5–28.2%; I^2^ = 93.4% [91.8–94.6%]). Overlapping 95% confidence intervals showed that the difference between the proportion of MRSA identified in studies from HICs and U/LMICs was not significant.

## Discussion

The microbiology of diabetic foot infection has been well characterised using classical microbiological and molecular techniques. This meta-analysis examined culture results from eligible studies to provide an overview of the microbiology of diabetic foot infections. A diverse range of bacterial genera were identified. A perennial challenge is the delineation of which microbe(s) are pathogenic and which are incidental. Molecular investigations have shown that diabetic foot infections often contain an array of organisms [29, 30]. The analysis of the microbiology of diabetic foot infections is further complicated by the potential for contamination of clinical samples by commensal bacteria. A high frequency of coagulase-negative Staphylococci was observed in this meta-analysis, which likely reflects a combination of sample contamination and genuine pathology caused by the introduction of commensals into tissues. Nonetheless, this meta-analysis clearly identified a high prevalence of bacterial species/genera classically associated with diabetic foot infection, e.g. *S. aureus*. The prevalence of MRSA identified by this meta-analysis (18.0%) matches closely with previous estimates [12, 31]. We acknowledge that there can be notable local variation in the microbiology of diabetic foot infections, and local microbiological knowledge must drive day-to-day clinical practice. However, macroscopic data such as this will be useful for prioritising targets for novel therapeutic interventions, for example bacteriophage therapy [32].

We used these data to investigate a previous assertion in the literature that Gram positive organisms might predominate in diabetic foot infections from more developed nations [28]. These data supported this correlation, with a significantly greater prevalence of Gram positive isolates among HICs and Gram negative isolates among U/LMICs. This held, although not always significantly, in general and with examination of the most commonly occurring Gram positive (*S. aureus*, *Streptococcus* spp., *Enterococcus* spp.) and Gram negative organisms (*Pseudomonas* spp., *E. coli*, *Klebsiella* spp., *Proteus* spp.). This may reflect differences in sanitation, hygiene or use of footwear [28, 33].

These data are limited by several factors. Firstly, to calculate the meta-analytic prevalence of each bacterial genera or species it was necessary to assume that all included studies had the same chance of detecting any given genera or species. In practice, this will depend on the skill, equipment and practices of the individual clinical microbiology laboratories. This will create underestimations of prevalence for bacterial genera or species that are uncommon or difficult to detect. Secondly, the majority of studies were conducted in U/LMICs (n = 43/112), notably India (n = 21/112). This may reflect negative publication bias among HICs, where such data may not be considered to be of sufficient interest for publication. This may create an over-representation of the prevalence of Gram negative organisms. Thirdly, the heterogeneity (I^2^) in the meta-analytic data was generally high, likely reflecting the multiple factors that would create different local microbiological profiles (e.g. patient demographics, circulating bacterial strains, local hygiene). Fourthly, this study may also be limited by not being pre-registered. Registration of reviews is a non-essential recommendation designed to encourage transparency, improve quality and reduce duplication. Pre-registration of reviews that are undertaken during student training or that are never completed is not recommended [34]. This study was conceived as a student project, many of which are not published, and the authors therefore decided it was inappropriate to register retrospectively. However, the authors are not aware of any similar studies underway and complied with the PRISMA statement throughout. Despite these limitations, these data provide a generally robust overview of the bacterial most frequently identified in diabetic foot infections.

## Conclusion

The microbiology of diabetic foot infections is diverse. Globally, *S. aureus* is the organism most commonly identified in diabetic foot infections, with MRSA representing 18.0% of *S. aureus*. There is a correlation between Gross National Income and diabetic foot microbiology, which likely reflects variations in sanitation. Knowledge of the microbiology of diabetic foot infections will help direct the development of novel therapeutics, such as bacteriophage therapy.

## Supplementary Information

**Supplementary file 1:** PRISMA checklist 

**Supplementary file 2:** Data extracted from included studies 

**Supplementary file 3:** Critical appraisal 

## Data Availability

Data sharing is not applicable to this article as no datasets were generated or analysed during the current study.

## Abbreviations

CIs: 95% Confidence intervals
MRSA: Methicillin-resistant *Staphylococcus aureus*
PRISMA: Preferred Reporting Items for Systematic Reviews and Meta-Analyses
